# Body mass index and dental caries in children aged 5 to 8 years attending a dental paediatric referral practice in the Netherlands

**DOI:** 10.1186/s13104-015-1715-6

**Published:** 2015-12-01

**Authors:** Maddelon de Jong-Lenters, Paula van Dommelen, Annemarie A. Schuller, Erik H. W. Verrips

**Affiliations:** TNO, Netherlands Organisation for Applied Scientific Research, Schipholweg 77-89, 2316 ZL Leiden, The Netherlands; Centre for Dentistry and Dental Hygiene (CTM), University Medical Center Groningen, Hanzeplein 1, 9713 GZ Groningen, The Netherlands; Department of Cariology Endodontology Pedodontology, Academic Centre for Dentistry Amsterdam, University of Amsterdam and VU University, Gustav Mahlerlaan 3004, 1081 LA Amsterdam, The Netherlands; Department of Preventive Dentistry, Academic Centre for Dentistry Amsterdam, University of Amsterdam and VU University, Gustav Mahlerlaan 3004, 1081 LA Amsterdam, The Netherlands

**Keywords:** Body mass index, Dental caries, Obesity prevention, Child

## Abstract

**Background:**

Obesity and dental caries are widely-recognised problems that affect general health. The prevention of both dental caries and obesity have proven very difficult: children and their parents may need professional support to achieve behaviour change. To find out whether both dental caries and overweight in childhood can be targeted using a common risk factor approach, it is necessary to establish whether the two diseases are indeed linked. The aim of the present study was therefore to use anthropometric data obtained professionally to investigate the association between Body Mass Index and dental caries experience in children aged 5–8 years receiving treatment in a referral centre for paediatric dental care in the Netherlands.

**Methods:**

Children’s dmft and dmfs scores were calculated using dental records and sociodemographic data were also extracted from these records. Dentists were trained to measure standing height and weight in a standardised way. Body Mass Index was calculated by dividing kilograms by height squared (kg/m^2^). Extended International (International Obesity Task Force) body mass index cut-offs were used to define ‘no overweight’ and ‘overweight’ (with the latter category including obesity).

**Results:**

No statistically significant differences were found between the mean dmft or dmfs scores of the two groups (overweight and non-overweight), even after correction for the effect of the potential confounders sex, socio-economic status and ethnicity. The percentage of caries-active children in the non-overweight group was almost the same as in the overweight group. No statistically significant differences were found.

**Conclusions:**

We hypothesised to find a positive association between body mass index and dental caries experience in children aged 5–8 years attending our practice. However, this study did not find a relationship of this kind. A common risk factor approach for the prevention of caries and overweight is therefore not supported by our study.

## Background

Obesity is a widely recognised problem [1–3]. In the Netherlands, the trend for obesity in the past 30 years has been upwards, with the population of the large cites being most affected [4, 5]. In recent years, however, the rise seems to be flattening out in city areas [6, 7]. Even so, overweight remains a major health concern since it has a major impact on general health and can induce chronic disease [8–10]. Moreover, these health effects become manifest not only in later life but also at an early age [11]. It has proven challenging to achieve the behaviour change needed to maintain or achieve a healthy weight [12]. This issue is therefore a major concern, particularly in children, since it has been suggested in the literature that childhood obesity is a predictor of obesity in later life [13, 14].

Dental caries still affects many schoolchildren, despite all the efforts made in prevention programmes. Recently, a prevalence of 41 % was reported for 5-year-olds in the Netherlands [15]. It is a disease that, even in the context of the preventive effect of using fluoride toothpaste, largely results from difficulties in managing a healthy lifestyle: the inadequate removal of dental plaque and the frequent intake of sugary foods and drinks [16].

The prevention of both dental caries and obesity has proven to be very difficult: children and their parents may need professional support to achieve behaviour change [17]. To find out whether both dental caries and overweight in childhood can be targeted with a common risk factor approach, it is necessary to determine whether the two diseases are indeed associated. Several studies have been conducted linking children’s dental caries experience to Body Mass Index (BMI), but the results are contradictory. In a recent systematic review, Hayden also concluded that the literature is inconclusive and that further analysis of this association and its confounding variables is needed [18]. On the one hand, studies by Gerdin et al. and Willershausen et al., Powell et al. and Yao et al. found that obesity or an unhealthily high BMI were linked to a higher number of caries lesions [19–22]. On the other, Sheller et al. found no association between BMI and dmft (decayed-missing-filled teeth) or the number of pulp-involved teeth [23], whereas Benzian et al. and Bafti et al. actually found an inverse relationship, reporting an association between underweight and a higher mean dmft, although it should be pointed out that these studies were not carried out in Western countries [24, 25].

In the Netherlands, dentistry consists of primary and secondary dental care. If the General Dental Practitioner (GDP) feels unable to deliver the care needed, referral to specialists in various areas is possible. These include dental surgery, endodontics and paediatric dentistry. One fairly large paediatric referral practice is ‘Cleyburch junior’, which is attended by children as young as 1 or 2 years old, as well as by adolescents. In addition to delivering treatment, this centre aims to teach children and their parents how to care for their own teeth and gums and, where appropriate, to cope with the problems they encounter in the dental surgery, all in line with their individual needs, capacities and skills. After treatment at the centre finishes, the children are referred back to the GDP.

Dental caries was found in 84 % of the children attending the centre. The mean dmft was 4.6 (SD ± 3.4) [26]. Furthermore, our dentists’ impression is that most of the families referred find that there are obstacles that work against their efforts to implement healthy behaviours in their daily routines. Studies have indicated that parental beliefs, parenting and family interaction are associated with dental caries [27], [28]. Parents are responsible for establishing and maintaining their children’s routines, such as fruit and vegetable intake, sugar snacking, physical activity and oral hygiene. Children’s behaviours, attitudes and social norms are moulded by modelling, specific parenting skills and, more extensively, by interactions between all family members [29]. It is important to establish healthy behaviours from early childhood onwards since unhealthy behaviours acquired early in life are difficult to change in adulthood [30]. Moreover, studies have demonstrated that a range of health-related behaviours such as dietary behaviours, physical activity and oral hygiene behaviours are clustered in individuals [31]. Our practice, with its high caries rates, provides an excellent opportunity to explore the association between caries and obesity. We first conducted a pilot study to explore the association between BMI and caries in a group of children aged 5–8 years (n = 247). The results showed the expected association: more caries lesions were found in overweight children [32]. However, the limitations of this pilot study were that anthropometric data were self-reported by parents, that the response rate was fairly low (56 %) and that potential confounders were not included in the analyses. Furthermore, because the lifestyle and family factors mentioned here have also proven to be important determinants of the development of overweight and obesity [33], we hypothesised that children in our practice were more prone to being overweight. The present study was therefore established to investigate, on the basis of anthropometric data obtained by professionals, the association between BMI and dental caries experience in children aged 5–8 years who were treated in our referral centre for paediatric dental care.

## Methods

Approval for this study was obtained from The Central Committee on Research Involving Human Subjects in the Netherlands (VU MEtc, nr 2012/393). Prior to every measurement, the parents of the selected child were asked for written informed consent.

### Study sample

The data for this study were collected in a referral centre for paediatric dental care in Noordwijk (Netherlands). The general statistics for the practice indicate that children are referred for various diagnoses, including Early Childhood Caries in very young children (22 %), congenital dental disorders (18 %), psychological problems (18 %), behaviour management problems (15 %), fear (14 %) and developmental problems (6 %). The ages of the children referred vary widely. For the purposes of this study, we selected all the children aged 5–8 who came for a regular check-up at our centre between January 2013 and July 2013. Every child was included only once: if they had more than one visit during the data-collection period, children were not included again. Children with diagnosed disorders in the emotional or behavioural field, children with special needs and newly referred children were excluded.

### Data collection

Before the regular check-up, we asked all children who met the selection criteria to participate in the study. Parents were informed about the purpose of the study and were asked to sign a written consent form, which also stated that they gave permission to use data from their child’s clinical health records for the purposes of this study. The treatment of the children was not affected in any way by a refusal to participate.

#### Caries experience

It is standard practice in the referral centre to update all personal dental health records in a standardised way every time patients visit the practice. Reasons for restoration or extraction are recorded. The diagnosis of dental caries is based on periodical clinical examinations supported by dental X-rays, mostly bitewings, whenever possible. Children’s dmft and dmfs (decayed-missed-filled surfaces) scores were calculated on the basis of these dental records. Patients’ dmft and dmfs scores are widely used outcome measures for the extent of caries experience in the primary dentition: it is the sum of decayed (d), missing (m) and filled (f) teeth (t) or surfaces (s). Missing teeth were not scored if they were absent due to dental trauma, hypomineralisation, agenesis or routine exfoliation; they were only scored if records indicated that they were extracted due to caries. In this case, only three surfaces per extracted element were recorded. We used the dental health data on the day of the measurement to compute dmft/s scores. In addition, caries activity was defined as a score of 0 (caries-free) or one or more surfaces/elements affected (caries-active). Secondary teeth were not scored.

#### BMI

Dentists in our practice were trained to perform the anthropometric measurements in a standardised way. They were conducted in the paediatric dentist’s treatment room. Children’s standing height was measured to the nearest full centimetre using a stadiometer (Seca); their weight was rounded off to the nearest 0.1 kilogramme using a calibrated scale (Seca, Model 877). During these measurements, the children wore light clothing but no shoes. Body Mass Index was calculated by dividing kilograms by height squared (kg/m^2^). We subsequently used extended International (IOTF) body mass index cut-offs to define ‘no overweight’ and ‘overweight’ (with the latter category including obesity) [34].

#### Sociodemographic characteristics

Our practice uses a protocol that involves the recording of the child’s age, sex and ethnicity, and the mother’s highest level of completed education. Ethnicity is defined on the basis of the mother’s country of birth: the Netherlands or elsewhere. There are three categories for the highest educational level of the mother: (1) no education or elementary school, (2) lower secondary education and (3) higher education or university. These categories defined the variable ‘education level of the mother’.

### Statistical analysis

The outcome variables were dmft scores, dmfs scores, caries activity and BMI. We used linear regression analysis to determine whether or not there was a link between dmfs/dmft and overweight, with overweight being used as the predictor. Logistic regression analysis was used to determine whether or not there was a link between overweight and caries activity. The results were then corrected for the influence of potential confounders, in this case sex, ethnicity and education level of the mother. A *p* value <0.05 was regarded as statistically significant.

## Results

### Study sample

The study sample consisted of 230 children (response rate 98 %). The mean age of the sample was 7.0 (SD ± 1.2), and 56.5 % were girls. The mean dmft was 4.2 ± 3.4; 19.6 % of the children were caries-free and 18.3 % were classified as overweight. Table 1 presents the findings and general characteristics of the sample studied. No statistically significant difference in the mean dmft or dmfs scores was found between the two groups (overweight and no overweight), even after correction for the influence of the potential confounders sex, SES and ethnicity (Table 2). The percentage of caries-active children in the non-overweight group was almost the same as in the overweight group. No statistically significant differences were found.

**Table 1:** Outcomes and general characteristics of the studied sample (n = 230)

	Mean ± SD
Age	7.0 ± 1.2
Dmft	4.2 ± 3.4
Dmfs	10.3 ± 9.3
	N (%)
Dental caries	
Caries-free	45 (19.6)
Caries-active	185 (80.4)
Overweight^a^	
No	188 (81.7)
Yes	42 (18.3)
Sex	
Girl	130 (56.5)
Boy	100 (43.5)
Education level (mother)^b^	
High	46 (21.4)
Medium	95 (44.2)
Low	74 (34.4)
Ethnicity^c^	
Native	192 (83.8)
Immigrant	37 (16.2)

^a^Including obesity

^b^Missing data for 14 children

^c^Missing data for 1 child

**Table 2:** Differences in level of caries between the overweight and no overweight group

Outcome	Overweight^b^	Overweight^b^	B (95 % CI)	Adj. B^c^ (95 % CI)
	No	Yes		
	Mean ± SD	Mean ± SD		
dmft	4.2 ± 3.5	4.2 ± 3.4	0.04 (−1.1, 1.2)	−0.38 (−4.5, 0.7)
dmfs	10.2 ± 9.4	10.9 ± 9.1	0.68 (−2.5, 3.8)	−0.46 (−3.5, 2.6)

	Percentage (n)	Percentage (n)	OR (95 % CI)	OR (95 % CI)
Caries activity^a^	80.3 (151)	81.1 (34)	1.04 (0.5, 2.4)	0.72 (0.3, 1.8)

^a^Caries-active (yes) or caries-free (no)

^b^Overweight includes obesity

^c^Adjusted for sex, ethnicity and age

## Discussion

We hypothesised to find a positive association between BMI and dental caries experience in the children aged 5–8 years attending our practice. However, our study did not find a relationship of this kind. Given this result, we conclude that being caries-active is not a predictor for being overweight, nor the other way around. Based on this study alone, one could say that the dental practice cannot play a role in preventing obesity, even as a way of screening children for overweight, at least not based on dental health records. Nevertheless, the literature does recommend a common risk factor approach to preventing caries and obesity since eating and drinking habits seem to be an overlapping element [35, 36]. However, despite the fact that different health-related behaviours have been found to cluster in individuals, different approaches are reported to be necessary for each required behaviour change. We know from the literature that these required behaviour changes have to be identified in detail and interventions are more effective if change objectives are subsequently specified per health related behaviour [31, 37, 38] The literature also tells us that conventional prevention methods -often focusing only on delivering knowledge about health behaviours—are largely ineffective [39]. Obviously, more tailored interventions are required. This fact has been worked out in the theory of motivational interviewing, which has potential to improving oral health behaviours, especially compared to conventional education [40]. In this theory the patient is led through four stages, in which barriers and facilitators experienced by the patient or the patients parents define a very personal preventive plan; based on intrinsic motivation. Meanwhile, the health professional is guiding the patient in implementing this plan, even in more difficult times [41]. Fisher Owens developed a model that suggested a wide range of underlying determinants of dental caries at the child, family and community levels [42]. We therefore believe that a paradigm shift is needed to acknowledge psychosocial factors as determinants of health. In the prevention and treatment of obesity, for example, successful interventions addressing general parenting have indeed been developed and mapped in a review by Gerards [43].

Furthermore, as mentioned above, the apparent multifactorial character of both overweight and caries means that it is important to consider different causes. Some causes—sound oral hygiene for example—affect only one disease. Other common causes are more complex: the frequency of the intake of sugary foods and drinks could be a cause of caries but the exact quantity per portion is less relevant for caries than it is for overweight. Parents in our practice say that some children are ‘bad eaters’: they mean that they have to encourage these children more and the result is a higher frequency of the intake of sugary foods or drinks, with small amounts being eaten or drunk on each occasion.

One of the strengths of this study was the very high prevalence of caries experience in our sample (80.4 %). The overall prevalence in 5-year-olds in the Netherlands is 41 % [15]. In theory, the high prevalence in our practice should have facilitated the confirmation of the hypothesis. However, a limitation was that all parents are referred to our practice by a GDP. Some more concerned parents asked for a referral letter themselves and, since these were probably mostly parents of caries-free children, this may have affected the profile of the caries-free group, making the comparison of the caries-free and caries-active groups less reliable. The prevalence of overweight children in our practice was also higher: 18.3 % as opposed to 13–15 % in the Dutch population [4]. This fact could be seen as support for the hypothesis that BMI will be higher in children with caries. The present study used a more refined method than the pilot study that was also conducted by the authors. Anthropometric measurements were taken professionally and the effect of potential confounders was taken into account. There was almost no selection bias since all the children attending the practice were asked to enter the study and only 2 % declined. Furthermore, highly reliable dental health records with caries diagnosis based on X-rays made the design very strong. Far more lesions are detected with X-rays [44]. In 87 % of our children, bitewings were used and they were not used only when restraint of the child would have been required otherwise. In this minority, overview radiographs were taken if possible. In a small number of cases, no radiographs were made because there was no indication.

As mentioned earlier, the findings of this study must be seen in the context of its limitations. The sample is not representative of the general population, as can be seen in the high caries rate. Another potential limitation is that overweight and obesity may become apparent at an older age. Furthermore, bitewings were not used for all children to determine caries experience and, in some cases, treatment of caries at the GDP (before referral) may have been missed. The latter two factors may have resulted in a lower mean dmft.

Studies by Gerdin et al. and Willershausen et al. did provide support for the positive association hypothesised here. However, both studies looked only at children aged 10 years old and measurements were taken at multiple moments in time [19, 20]. As mentioned above, it is therefore reasonable to speculate that overweight is usually revealed at a higher age. Powell et al. also found a positive association when looking at younger children but his sample consisted only of children treated under general anaesthesia [21]. By contrast, a number of studies have shown that more caries experience was associated with being underweight. These studies found a very high caries rate: the authors saw this high caries prevalence and the low restoration rate as an indicator of pulp involvement and therefore a failure to thrive in general [24, 25].

The main reason for conducting this study, backed up by the observation that parents play an essential role in maintaining healthy behaviours in children, was the hypothesis that both obesity and dental caries can be targeted by a common risk factor approach. In our practice, parents encountered obstacles to the implementation of these behaviours, confirming suggestions found elsewhere in the literature relating to both obesity and caries [45, 46]. Improvements in dental health and behaviours must begin, however, with dental professionals wanting parents and children to succeed in prevention and recognition of the need to work together with all health professionals. Moreover, dental professionals will have to acknowledge the need to go beyond health education based on knowledge only (one-way traffic) and to start exploring what happens in the home in order to enhance the probability of active behaviour change.

## Conclusions

Although it was hypothesised to find a positive association between BMI and dental caries experience in children aged 5–8 years attending our practice, our study did not find evidence of that relationship. A common risk factor approach to the prevention of caries and overweight was not therefore supported by our study.

## Abbreviations

IOTF: International Obesity Task Force
dmft: decayed-missing-filled teeth
dmfs: decayed-missing-filled surfaces
BMI: body mass index
GDP: General Dental Practitioner
